# Evaluating the Clinical Performance of a Novel, Precision Oral Appliance Therapy Medical Device Made Wholly From a Medical Grade Class VI Material for the Treatment of Obstructive Sleep Apnea

**DOI:** 10.7759/cureus.50107

**Published:** 2023-12-07

**Authors:** Edward Sall, Kent Smith, Aditi Desai, John A Carollo, Mark T Murphy, Sung Kim, Leonard A Liptak

**Affiliations:** 1 Otolaryngology, Crouse Irving Memorial Hospital, Syracuse, USA; 2 Sleep Medicine, Star Sleep & Wellness, Frisco, USA; 3 Sleep Medicine, London Bridge Hospital, London, GBR; 4 Dental Sleep Medicine, Dental Sleep Medicine of New Jersey, Florham Park, USA; 5 Dental Sleep Medicine, Funktional Sleep, Rochester Hills, USA; 6 Sleep Medicine, ProSomnus Sleep Technologies, Pleasanton, USA

**Keywords:** osa treatment, upper airway collapsibility, snoring, precision oral appliance therapy, sleep disorderd breathing, mandibular advancement devices, hypoglossal nerve stimulator, oral appliance therapy, continuous positive airway pressure (cpap), obstructive sleep apnea (osa)

## Abstract

Objective

The objective of this study is to evaluate the clinical performance of a novel, precision, oral appliance therapy (OAT) medical device made entirely from a US Pharmacopeia (USP) medical grade class VI qualified material for the treatment of obstructive sleep apnea (OSA).

Methods

This was a multi-center, single-arm, chart-based, retrospective study of 91 patients diagnosed with OSA, treated utilizing a novel, precision, OAT medical device. Performance criteria were overall efficacy (reduction of OSA events to less than 10 per hour); efficacy for patients with severe OSA (reduction of OSA events to less than 20 per hour and a 50% improvement); and compliance (the rate of continuation of treatment after at least a one-year follow-up, or, conversely, the rate of discontinuation of treatment due to material-related adverse events or side effects after one year).

Results

Eighty-nine percent of all subjects diagnosed with all levels of OSA severity were successfully treated to an apnea hypopnea index (“AHI”) < 10 events per hour. Ninety-eight percent of subjects diagnosed with mild to moderate OSA were successfully treated to an AHI < 10. Eighty percent of subjects with severe OSA, without screening or excluding subjects for airway collapse profile, were successfully treated to an AHI < 20 with a 50% improvement in AHI. After a minimum one-year follow-up period, 96% of patients were confirmed to remain in active treatment. No subjects were reported to discontinue treatment due to adverse events or side effects.

Conclusions

This novel, precision OAT medical device made from the USP Class VI qualified material demonstrated efficacy and safety for the treatment of patients with OSA.

## Introduction

Clinical practice guidelines encourage clinicians to weigh adverse event risks when selecting a therapy and medical devices for the treatment of patients with obstructive sleep apnea (OSA) [1-4]. The US Food and Drug Administration (FDA) defines adverse events as undesirable experiences, including side effects, that should be reported when the outcome is death, life-threatening, hospitalization, disability, required intervention, or a serious medical event [5]. Adverse events, including side effects, are associated with lower rates of therapy compliance for the treatment of OSA [6-8].

It is thought that many adverse event reports in the FDA Manufacturer and User Facility Device Experience (MAUDE) database for continuous positive airway pressure (CPAP), hypoglossal nerve stimulation (HNS), and oral appliance therapy (OAT) devices, ranging from device degradations to allergic reactions, may be related to materials [6,8,9]. For example, an estimated 8% to 19% of adults have a nickel allergy [10]. Nickel is a material commonly used in the construction of the fixed mechanical hinge components of several OAT devices which allows for titration of the oral appliance [11].

The purpose of this study is to evaluate the clinical performance of a novel, custom, precision, titratable, OAT device made entirely from a medical grade USP-qualified class VI material. Class VI is the most stringent designation of material biocompatibility according to USP, consisting of six tests utilizing the International Organization for Standards (ISO) 10993-10 and 7405 criteria. Materials that pass each of these six tests are qualified as USP medical grade Class VI [12].

What is precision OAT? Precision OAT consists of patient-specific treatment plans, patient-specific oral appliance therapy medical devices, and patient-specific titration options that are designed based on the unique upper airway and dental anatomy of each patient [13,14]. Digital manufacturing technologies are utilized to ensure that each precision, patient-specific oral appliance therapy device is capable of repositioning, stabilizing, and titrating the mandible within < 1 mm of the therapeutic position associated with reducing upper airway collapse [15]. 

In contrast, traditional OAT utilizes impression materials for capturing patient dental anatomy records, does not consistently use instruments or protocols for determining nor capturing the therapeutic mandibular position, and a laboratory, artisanal, fabricated OAT device that repositions, stabilizes, and titrates the mandible with quality of > 5mm of error relative to the therapeutic mandibular position. Typically, traditional OAT devices are made from cold-cured, dental laboratory materials that often utilize metal components for the titration mechanism and laminate materials as device liners [11,16].

## Materials and methods

Study design overview

This multi-center, single-arm study uses a retrospective, chart-based, methodology was used to evaluate the treatment efficacy and compliance for this novel, precision OAT medical device. 

Population

Patients enrolled in this study were referred to dental sleep medicine therapy centers for treatment of OSA utilizing OAT. Four centers, Star Sleep & Wellness, Frisco, USA, London Bridge Hospital, London, GBR, Dental Sleep Medicine of New Jersey, Florham Park, USA, and Funktional Sleep, Rochester Hills, USA, participated in this study. Each of these centers has experienced dental sleep medicine therapy providers, qualified by their respective professional academies [17,18]. 

Intervention

**Figure 1:**
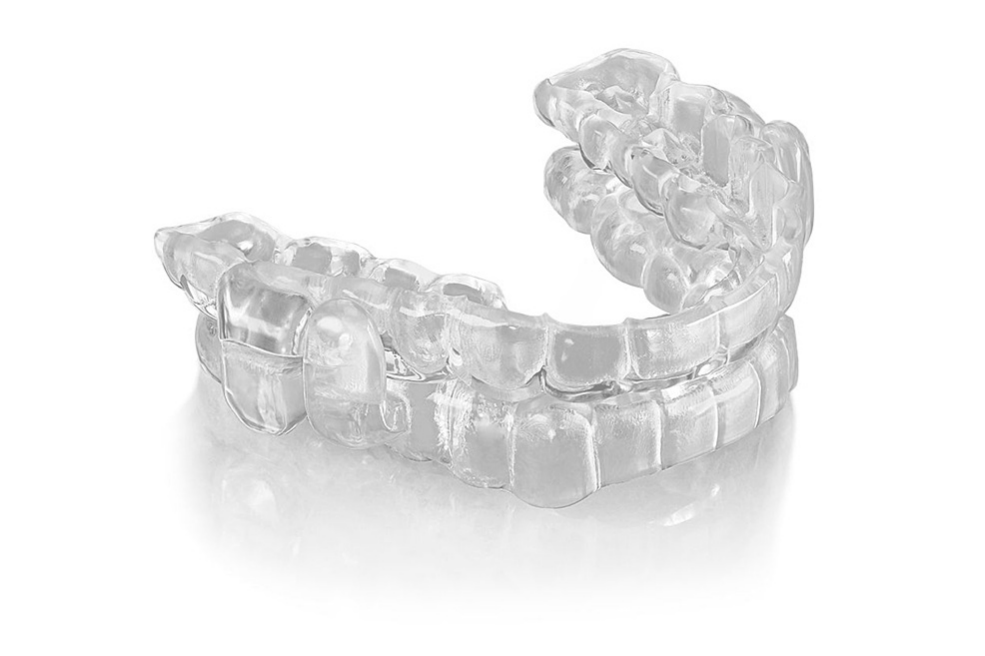
ProSomnus EVO OAT Device Image credit: ProSomnus Sleep Technologies OAT: Oral appliance therapy

A novel, custom, iteratively titratable, precision, FDA-cleared OAT device made entirely (each arch component of the device is monolithic) from a medical grade USP class VI qualified material (ProSomnus EVO, Pleasanton, CA, USA, Figure 1) was utilized for the treatment of patients enrolled in this study. Precision OAT incorporates digital technologies to more accurately incorporate each patient’s upper airway and dental anatomy into designing the treatment plan and oral appliance therapy medical device for each patient to reduce upper airway collapsibility [11,13-15]. Patients were titrated based on symptoms. Upon symptom alleviation, patients were referred for testing to confirm the efficacy of the treatment. 

USP Class VI is the highest designation of material biocompatibility for medical devices [12]. USP Class VI qualification consists of six tests utilizing the International Organization for Standards (ISO) 10993-10 and 7405 criteria. The six tests include skin sensitization, acute oral mucosa irritation, acute systemic toxicity, in vitro cytotoxicity by agar diffusion method, in vitro cytotoxicity by filter diffusion method, and in vitro cytotoxicity by elution method. The novel OAT device, the test item, was subjected to USP Class VI tests by a qualified, independent, laboratory (Bioneeds, Karnataka, India). The novel OAT device passed each of the six tests. See Table 1 for additional details.

**Table 1:** ISP Class VI Tests and Conclusions for the Novel OAT Device (Data for Table Provided by Bioneeds, Karnataka, India)

No.	Test	Standard(s)	Report No.	Test Conclusion
1	Skin Sensitization	ISO 10993-10	BIO-ATX 1856	The polar and non-polar extracts of the test item was found to be “non-sensitizer” to the skin.
		ISO 7405		
2	Acute Oral Mucosa Irritation	ISO 10993-10	BIO-ATX 1857	The evaluation of biological response to application of polar and non-polar extract of the test item was considered as a non-irritant.
		ISO 7405		
3	Acute Systemic Toxicity	ISO 10993-11	BIO-ATX 1858	The polar and non-polar extracts of the test item when administered through intravenous and intraperitoneal routes respectively at a dose volume of 50 mL/kg body weight did not reveal any systemic toxicity.
		ISO 7405		
4	In Vitro Cytotoxicity by Agar Diffusion Method	ISO 10993-5	BIO-GNT 831	The test item was found to be “non-cytotoxic” to the subconfluent monolayer of L-929 fibroblast cells tested under laboratory conditions.
		ISO 7405		
5	In Vitro Cytotoxicity by Filter Diffusion Method	ISO 10993-5	BIO-GNT 832	The test item was found to be “non-cytotoxic” to the subconfluent monolayer of L-929 fibroblast cells tested under laboratory conditions.
		ISO 7405		
6	In Vitro Cytotoxicity by Elution Method	ISO 10993-5	BIO-GNT 833	The test item was found to be “non-cytotoxic” to the subconfluent monolayer of L-929 fibroblast cells tested under laboratory conditions.
		ISO 7405		

Comparison

Comparison criteria for this pilot study consisted of no treatment (baseline) and performance goals established in medical guidelines for the treatment of obstructive sleep apnea [1-4]. Home sleep testing was used to determine the baseline and residual (with treatment) AHI events per hour for each patient. 

Outcomes

There were four performance goals for this pilot study:

1. Determine the mean percent improvement in Apnea-Hyponea Index (AHI), a count of Apnea-hypopnea events per hour, by comparing the baseline (no treatment) AHI with the residual (with OAT) AHI data for the study population.

2. Quantify the percentage of patients that improved their AHI below 10 events per hour, in total. 

3. Evaluate and stratify therapeutic success by OSA severity (mild, moderate, and severe). Quantify the percentage of patients with mild to moderate OSA that demonstrated an improvement in their AHI below 10 events per hour. Calculate the percentage of study patients with baseline severe OSA (AHI > 30 events per hour) that improved to a residual AHI of less than 20 events per hour and a 50% improvement in events, Sher’s success criteria for therapeutic success [19]. Sher’s criteria for therapeutic success are an endpoint recently used to evaluate the success of treating severe OSA patients with HNS [20].

4. Evaluate the rate of continuing use of the therapy, defined as the percentage of patients continuing to use the therapy after a minimum of one-year follow-up, and specifically analyze the number of patients who discontinued treatment due to material-related problems. 

Treatment efficacy, outcomes #1-#3, were determined by comparing the baseline (pre-treatment) and residual (with treatment) sleep apnea events per hour (AHI) from patient charts. Compliance, outcome #4, was determined by evaluating the number of patients who reported continued usage of the novel, precision OAT device after no less than one year of follow-up. Both the efficacy and compliance, continuing usage, data were compiled retrospectively from patient charts. 

## Results

The study population consisted of 91 patients diagnosed with OSA. The mean age was 53.3 +/- 11.4 years. Sixty-seven percent of patients were male; 33% were female. The mean, baseline AHI was 23.9 +/- 16.7 events per hour. The maximum, baseline patient AHI was 116 events per hour. The minimum, baseline patient AHI was 6 events per hour. Thirty (33%), 36 (40%), and 25 (27%) patients were classified with mild, moderate, and severe OSA, respectively. 

Outcome #1: The mean improvement in AHI events per hour relative to baseline was 76.7% with the novel OAT intervention. 

Outcome #2: Eighty-nine percent of the patients enrolled in this study were efficaciously, successfully, treated, achieving the AHI < 10 performance goal.

Outcome #3: Ninety-eight and one-half percent of the sixty-six patients diagnosed with mild to moderate OSA were successfully treated to the performance goal of AHI < 10. Of the twenty-five patients classified with severe OSA, 80% successfully demonstrated efficacy with the novel OAT device according to the Sher’s criteria performance goal of AHI <20 and 50%.

A paired t-test was used to evaluate the statistical significance of the residual AHI relative to the baseline AHI. All results were statistically significant at a P < 0.0001 level with a 95% confidence interval (see Table 2).

**Table 2:** Tests of Statistical Significance

	Sample Size	Baseline AHI	Residual AHI	Significance Level
Total Sample	91	23.9 +/- 16.7	5.6 +/- 5.3	P < 0.0001
Mild and Moderate OSA	66	16.2 +/- 6.1	3.8 +/-2.4	P < 0.0001
Severe OSA	25	44.4 +/-18.7	10.2 +/-7.8	P < 0.0001

Outcome #4: After a minimum one-year follow-up period, 96% of patients, 87 of 91 patients, reported to be continuing users of the novel, precision, OAT device. Four patients could not be confirmed as continuing users. Three patients were lost to follow-up. The fourth patient reverted to CPAP. No patients discontinued treatment with the novel OAT due to material-related issues. 

## Discussion

This multi-center, single-arm, chart-based, retrospective study indicates that treating OSA patients with the novel, precision, OAT device made from USP Class VI qualified material is efficacious and associated with durable, continuing use after a minimum one-year follow-up. 

The percent improvement in AHI was 76.6% with the novel OAT device, from a mean, pre-treatment, baseline AHI of 23.9 events per hour to a mean, with treatment, residual AHI of 5.6 events per hour. This level of improvement agrees with what has been reported in other studies for precision OAT [14,21-24]. The efficacy reported in this study is also directionally better than what has been reported for traditional oral devices [14,25,26]. 

Eighty-nine percent of the total study population experienced therapeutic efficacy, successfully satisfying the AHI performance goal of less than 10 events per hour. 

Stratification by OSA severity level can be a useful tool for assisting healthcare providers in selecting the optimal treatment for each patient. For this study, stratification of the study population reveals that 98% of mild and moderate OSA patients achieved the AHI < 10 performance goal. The implication of these results is that intervention with this novel OAT demonstrates efficacy for patients with mild and moderate OSA and may be a viable alternative to patients who refuse or fail CPAP therapy, or, potentially, as frontline therapy or combination therapy. 

Standardizing the definition of efficacy, the probability that a patient will be successfully treated by an intervention, is also an important tool for helping healthcare providers select the optimal therapy for each patient. Although this is not a head-to-head study, Sher’s criteria of efficacy, AHI < 20 and a 50% improvement over baseline, was selected to facilitate an understanding of performance relative to other therapies, such as hypoglossal nerve stimulation which uses Sher’s criteria, for patients diagnosed with severe OSA. 

In this study, 80% of all severe OSA patients, with no exclusion criteria for concentric airway collapse profiles, were successfully treated with non-invasive, oral appliance therapy with the novel precision oral appliance therapy device successfully demonstrated the efficacy of AHI < 20 events per hour and a 50% improvement. The relevance of concentric collapse is that it is a contraindication for HNS therapy. Research has also identified concentric collapse as an adverse phenotype; patients with a concentric collapse profile are less likely to respond to treatment [27]. Thus when efficacy data is reported for HNS procedures, it typically excludes patients with concentric collapse. Invasive HNS treatments are typically associated with 66% to 69% efficacy relative to Sher’s criteria, even with the exclusion of patients with concentric collapse airway profiles [20,28]. This finding suggests that physicians and patients might consider precision OAT prior to undergoing HNS surgery, or in combination with HNS or CPAP treatment. 

OSA is a chronic disease that afflicts an estimated one billion people worldwide [29]. Untreated OSA is associated with significant medical consequences and economic costs [30-33]. Treatment compliance and continuing use are essential for the efficient treatment of such a large population of at-risk patients due to the dose dependency between treatment compliance and health benefits [1-4,15,2-24,33,34]. Furthermore, it is thought that side effects and adverse events are associated with decreased rates of compliance, adversely impacting the durability, effectiveness, and efficiency of treatment which establishes the rationale for utilizing a precision OAT medical device that is made wholly from a USP medical grade Class VI material and delivered by qualified dental sleep medicine centers [1-4,6,8,35,36].

For this study, 96% of patients were continuing users of the treatment after a minimum one-year follow-up. This finding is different from investigations that utilized traditional-style OAT devices that report a higher rate of discontinuation of therapy due to various side effects [34,36,37]. In this study, all patients were confirmed as continuing users of the treatment with a minimum one-year follow-up. 

There are several additional limitations to this study. The first is the study design. This is a retrospective, single-arm, chart-style analysis. These findings may establish an impetus for a prospective, randomized, controlled study.

Another limitation is generalizability. This study utilized one type of precision oral appliance therapy device. This study utilized only dental sleep medicine providers who are qualified and experienced with precision oral appliance therapy. Thus, the results might not be applicable to other types of non-precision oral appliance therapy devices made wholly from non-USP Class VI materials or less qualified, less experienced dental sleep medicine providers. It is estimated that less than 2% of dentists are qualified, experienced, and credentialed dental sleep medicine providers. As such, these results might not be generalizable to healthcare providers who are less qualified, experienced, and credentialed in dental sleep medicine. Lastly, the four centers employed different testing regimens: timings, protocols, and brands of diagnostic devices used for testing, although each center attempted to match pre / post-testing methods and brands. 

This investigation establishes a foundation for further research. A prospective, randomized, controlled trial comparing the efficacy, compliance rates, consistency, and durability of this novel, precision OAT device made from Class VI qualified material with either traditional OAT devices, CPAP, or HNS interventions could be instructive. Furthermore, a future study can account for additional precision OAT device titrations. The residual efficacy reported in this study reflects results of tests conducted after symptom alleviation. Patients may have undergone additional titrations, potentially resulting in improved outcomes that may not be reflected in this study.

## Conclusions

In this investigation, a novel, precision, OAT device made from the USP Class VI qualified material was associated with efficacious treatment, consistency, and durable compliance. Patients diagnosed with mild and moderate OSA who were treated with the novel, precision OAT device demonstrated efficacy and continuing use rates that suggest consideration as an alternative therapy for CPAP. Severe OSA patients treated with this novel, non-invasive, OAT device demonstrated efficacy, using Sher’s criteria, on par with what has been reported for hypoglossal nerve stimulation, even when including concentric collapse patients in the patient population for the novel OAT. 
